# Chimiothérapie du cancer du sein au cours de la grossesse: à propos d´un cas

**DOI:** 10.11604/pamj.2021.38.255.18879

**Published:** 2021-03-11

**Authors:** Henintsoa Murielle Rakotomalala, Andriatsihoarana Voahary Nasandratriniavo Ramahandrisoa, Malala Razakanaivo, Ny Ony Andrianandrasana, Florine Rafaramino

**Affiliations:** 1 Faculté de Médecine d’Antananarivo, Antananarivo, Madagascar,; 2 Service d’Oncologie, Centre Hospitalier Universitaire Joseph Ravoahangy Andrianavalona, Antananarivo, Madagascar

**Keywords:** Cancer du sein, chimiothérapie, grossesse, réunion de concertation pluridisciplinaire, à propos d’un cas, Breast cancer, chemotherapy, pregnancy, multidisciplinary consultation, case report

## Abstract

La prise en charge d´un cancer du sein associé à une grossesse constitue un défi pour les médecins qui vient souvent du désir de la mère de mener à terme la grossesse malgré la nécessité d´une chimiothérapie. Une femme de 37 ans, multipare, enceinte à 20 semaines d´aménorrhée (SA) et 4 jours a été hospitalisée pour dyspnée NYHA IV. La patiente présentait un syndrome d´épanchement pleural liquidien gauche de grande abondance et une mastite bilatérale. Le diagnostic de cancer du sein métastatique était retenu par un examen cytologique du liquide pleural et une cytoponction mammaire révélant un carcinome galactophorique. La patiente a bénéficié d´un drainage pleural avec amélioration de la dyspnée mais le liquide pleural restait intarissable. Après discussion en réunion de concertation pluridisciplinaire (RCP), un traitement spécifique du cancer était impératif. Cinq cycles de chimiothérapie du protocole associant le 5-Fluoro-uracile, Epirubicine et Cyclophosphamide avaient été réalisés après le consentement du couple. L´épanchement pleural avait fortement diminué après la deuxième cure. En concertation avec l´obstétricien, la chimiothérapie avait été arrêtée un mois avant la 37^e^semaine d´aménorrhée. L´évolution de la grossesse était favorable, l´accouchement est dirigé après rupture des membranes avec issu par voie basse d´un bébé né à terme avec une bonne adaptation néonatale. A un an du suivi, la mère est toujours en cours de chimiothérapie et le bébé est en bonne santé. Plusieurs paramètres sont à considérer lors de l´administration des antinéoplasiques d´où l'intérêt d'une surveillance fœtale et obstétricale rapprochée. Une approche multidisciplinaire est recommandée pour la décision thérapeutique et le suivi.

## Abstract (en)

The management of breast cancer during pregnancy is a challenge for physicians due to mother´s desire to carry the pregnancy to term despite the need for chemotherapy. This study reports the case of a 37-year-old multiparous woman at 20 weeks and 4 days of amenorrhea (WA). She was hospitalized for dyspnoea (stage IV according to New York Heart Association (NYHA) classification). The patient had a syndrome of heavy left pleural effusion and bilateral mastitis. The diagnosis of metastatic breast cancer was retained based on cytological examination of pleural fluid and breast cytoponction revealing galactophoric carcinoma. The patient underwent pleural drainage with improvement of dyspnea but pleural fluid continued. After multidisciplinary consultation (MC), specific treatment of cancer was necessary. Five cycles of epirubicin- cyclophosphamide-5-FU-based chemotherapy was performed after the couple provided consent. Pleural fluid diminished significantly after the second cycle of treatment. After consultation with the obstetrician, chemotherapy was interrupted one month before the 37^th^ week of amenorrhea. Pregnancy evolved favorable, vaginal birth was managed following rupture of membranes at term with good neonatal adaptation. After one-year follow-up, the mother was still on chemotherapy and the baby was in good health. Several parameters should be considered before the administration of antineoplastic agents, hence the role of early fetal and maternal monitoring. Multidisciplinary approach is recommended to support therapeutic decision and follow-up.

## Introduction

Le cancer du sein associé à une grossesse est un cancer du sein diagnostiqué au cours de la grossesse ou au cours des douze mois post-partum [1]. C´est le cancer le plus rencontré au cours de la grossesse [2] avec une incidence de 1 sur 3000 grossesses [3] et constitue 0,4% des cancers du sein diagnostiqués chez les femmes entre 16 à 49 ans [4]. Les cancers du sein associés à une grossesse sont le plus souvent diagnostiqués à un stade avancé [1, 5]. Leur prise en charge constitue un défi pour les médecins. La décision de débuter une chimiothérapie est difficile à prendre du fait des risques materno-foetaux [6]. Nous rapportons un cas de cancer du sein associé à une grossesse qui a bénéficié d´une chimiothérapie au cours de la grossesse, avec bon issu de la grossesse et bonne évolution de la mère.

## Patient et observation

Une femme de 37 ans enceinte de 20 semaines d´aménorrhées (SA) et 4 jours a été admise au service des urgences pour dyspnée d´installation progressive mais d´aggravation aiguë en intensité de NYHA II à IV, 3 jours avant son admission. Dans ses antécédents gynécologiques, sa ménarche était à l´âge de 14 ans, elle est multipare G4P3A0, avec 2 enfants vivants allaités aux seins, l´âge de la première grossesse était à 23 ans, elle n´a jamais eu de contraception. Il n´y avait pas d´antécédents de cancer dans la famille. Deux ans auparavant, elle avait palpé mais négligé un nodule au niveau du quadrant supéro-externe du sein gauche.

**Figure 1:**
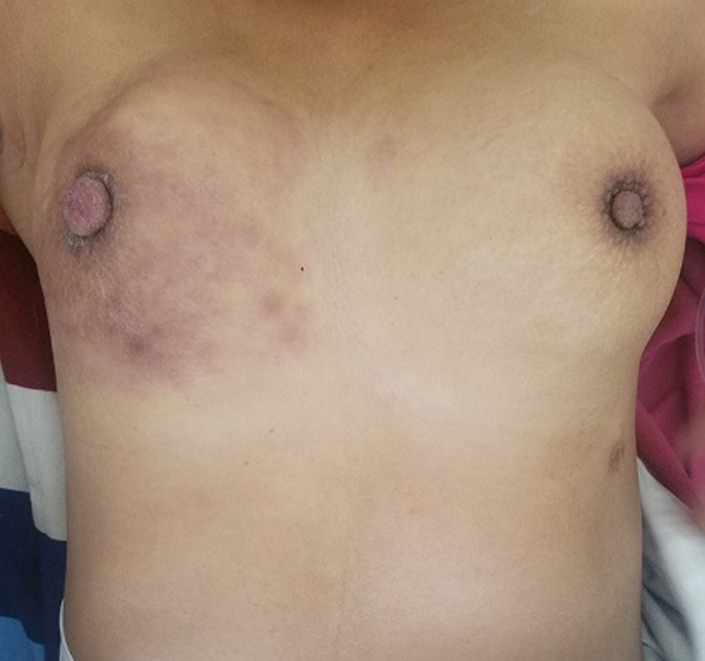
masses mammaires bilatérales

**Figure 2:**
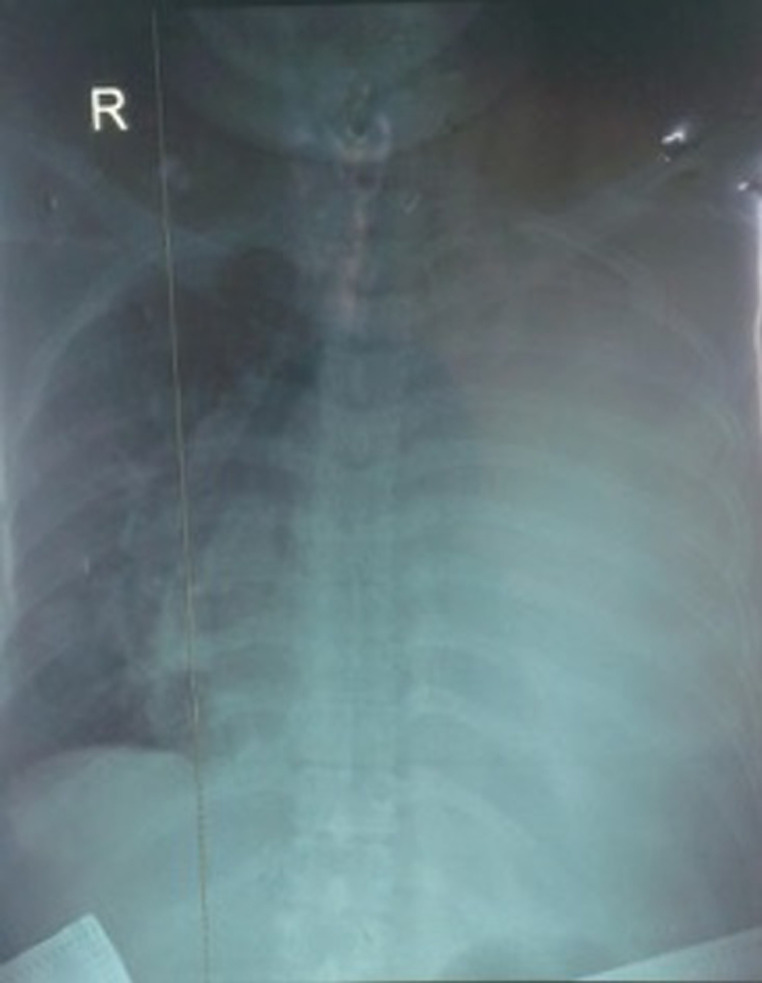
radiographie du thorax de la patiente

A l´examen, la patiente était agitée. Elle présentait un syndrome d´épanchement pleural liquidien de grande abondance à gauche. Elle présentait également une mastite bilatérale (Figure 1) et des adénopathies axillaires bilatérales, fermes, mobiles et non douloureuses. L´examen neurologique, cardio-vasculaire étaient sans particularités. L´examen obstétrical fait était normal. Une radiographie du thorax avec port de tablier plombé a été faite objectivant une opacité de tout le champ pulmonaire gauche, un médiastin refoulé vers la droite et des nodules pulmonaires droits en lâchée de ballon (Figure 2). L´échographie mammaire révélait des seins hétérogènes avec forte suspicion d´infiltration tumorale maligne et des adénopathies axillaires d´allure secondaire. Une cytoponction mammaire indiquait un carcinome galactophorique.

La patiente a bénéficié d´un drainage pleural en urgence, dont la cytologie du liquide pleural était en faveur d´une localisation secondaire d´un carcinome galactophorique. Au bout de 10 jours, le liquide pleural restait intarissable. Une RCP réunissant les oncologues, les chirurgiens, les obstétriciens et les néonatologues ont décidé de débuter en urgence une chimiothérapie avec surveillance rapprochée de la grossesse. Avec le consentement du couple, la patiente a reçu 5 cycles de chimiothérapie du type FEC 75mg (5-Fluoro-Uracile 750mg, Epirubicine 75mg, Cyclophosphamide 750 mg), bien tolérés dans l´ensemble. Au bout de la 2^e^ cure, il y avait une diminution nette de la pleurésie et une diminution du volume des seins. La patiente est entrée spontanément en travail, elle présentait une rupture des membranes, le travail était dirigé; elle a accouché par voie basse à 38 SA 4 jours d´un bébé né vivant sans malformations visibles, de genre masculin, APGAR 10 à M5 avec un poids de naissance de 2900g. A 1 an du suivi, le bébé est en bonne santé, il présente un bon développement psychomoteur et un bon développement staturo-pondéral; la mère est en cours de chimiothérapie sans progression.

## Discussion

Le cancer du sein associé à une grossesse est rare, il représente environ de 0,2 à 1% de tous les cancers du sein [3]. C´est une grossesse à haut risque. L´association de cancer du sein bilatéral et grossesse reste exceptionnel [6]. Quinze pourcent des femmes de moins de 40 ans ayant un cancer de sein sont enceintes lors du diagnostic [3]. Cette survenue du cancer du sein à un âge jeune, explique la coexistence d´une grossesse [6] mais aussi leur nombre plus fréquents qu´il y a 20 ans passés [7]. A notre connaissance, notre cas représente le premier cas de cancer du sein bilatéral associé à une grossesse rapporté à Madagascar.

Le cancer du sein associé à une grossesse est le plus souvent diagnostiqué à un stade avancé que chez les femmes non enceintes [1]. Les études récentes démontrent que le mauvais pronostic est aussi lié à un retard au diagnostic et au report traitement en vue de garantir une bonne issue de la grossesse [3, 7, 8]; et parfois à un profil immunohistochimique particulier de la patiente rendant la tumeur plus agressive, notamment le dit « triple négatif » [6, 9]. Pour notre patiente, le diagnostic se faisait au stade métastatique à 20 SA et 4 jours. La prise en charge des pathologies malignes chez les femmes enceintes constitue un défi tant pour les obstétriciens, les oncologues et les néonatalogues [1]. L´interruption de la grossesse n´améliore pas le pronostic de la mère, sa poursuite serait même corrélée à une meilleure survie maternelle [6]; ce malgré qu´elle est seulement considérée au premier trimestre lorsque la nécessité de débuter d´un traitement est une urgence [2, 10].

Le pronostic de la mère est mauvais [6]. Les bénéfices pour la mère doivent être prises en compte et balancées avec les effets d´une chimiothérapie sur le fœtus [11]. Le premier trimestre est le moment le plus critique par rapport à des effets tératogéniques [11], les risques potentiels d´une chimiothérapie sont l´avortement et les malformations congénitales [2]; et au second et troisième trimestre, les risques les plus décrits sont les faibles poids à la naissance et la prématurité [12]. Toutes les chimiothérapies sont potentiellement tératogènes, elles sont contre-indiquées au premier trimestre de grossesse mais elles sont acceptables durant le second et le troisième trimestre [3, 8]. Le fait de différer le traitement favorise le risque carcinologique de poursuite évolutive, de métastase transplacentaire [1, 6]; et de décès de la mère [1, 7].

Basé sur les recommandations de l´ESMO (European Society of Medical Oncology), le traitement suit celui de la femme non enceinte prenant en considération l´âge gestationnel au diagnostic et le terme. Les premiers choix sont les molécules à base d´anthracyclines [2]. Plusieurs cas et séries de cas de traitement de la femme enceinte avec une polychimiothérapie incluant l´Epirubicine n´ont pas montré de cardiotoxicité fœtale; les cas décrits sont limités [11]. Quand l´utilisation d´anthracyclines est contre-indiquée ou s´il n´y a pas de réponse, les taxanes seront adoptés, de préférence le Paclitaxel [2]. Les cas décrits par Ye en 2017 en Chine démontrent la bonne tolérance d´une chimiothérapie combinée de Epirubicine-Paclitaxel tant pour la mère que pour le fœtus [8]. L´entrée en travail peut arriver spontanément après la 34^e^ semaine d´aménorrhée (SA), l´administration de traitement général devrait donc être arrêtée avant la 33^e^ SA pour éviter un accouchement dans la période nadir [2]. Ce qui a été faite pour notre patiente. L´utilisation de Tamoxifène est contre-indiquée durant la grossesse et l´utilisation de Trastuzumab doit être différée après l´accouchement [2, 13].

Amant *et al*. en 2015 ont comparé des enfants ayant été exposés en anténatal à une chimiothérapie à d´autres enfants; leur résultat a montré que ces enfants avaient un développement normal; la chimiothérapie n´a pas d´effets néfastes sur le développement psychomoteur et le développement staturo-pondéral ni sur la fonction cardiaque de ces enfants [12]. Notre bébé se trouve en bonne santé. Un plan personnalisé de soins décidé en réunion de concertation pluridisciplinaire prenant en compte plusieurs paramètres tels que l´âge gestationnel, le stade du cancer, et la préférence de la patiente est d´une importance majeure dans la prise en charge du cancer du sein associé à une grossesse [4].

## Conclusion

Après le premier trimestre de grossesse, une chimiothérapie du cancer du sein associé à une grossesse qui est bien tolérée et avec bonne issue de la grossesse est possible. Certes il y a des recommandations pour le traitement du cancer du sein chez la femme enceinte mais il n´y a pas de consensus; tous les cas doivent être discutés et présentés devant une réunion de concertation multidisciplinaire pour maximiser les bénéfices de la mère et minimiser les risques pour le fœtus.
